# Identification of *Bcl2* as a Stably Expressed qPCR Reference Gene for Human Colon Cancer Cells Treated with Cottonseed-Derived Gossypol and Bioactive Extracts and Bacteria-Derived Lipopolysaccharides

**DOI:** 10.3390/molecules27217560

**Published:** 2022-11-04

**Authors:** Heping Cao, Kandan Sethumadhavan

**Affiliations:** United States Department of Agriculture, Agricultural Research Service, Southern Regional Research Center, 1100 Allen Toussaint Boulevard, New Orleans, LA 70124, USA

**Keywords:** bioactivity, colon cancer cell, cottonseed extract, gene expression, gossypol, lipopolysaccharides, quantitative real-time PCR, reference gene

## Abstract

Cottonseed contains many bioactive molecules including plant polyphenols. Cottonseed value might be increased by providing high-value bioactive polyphenols for improving nutrition and health. However, there was a lack of molecular evidence for cottonseed bioactivity in mammalian cells. One widely used method for evaluating the bioactivity of natural products is quantitative real-time-PCR (qPCR). The selection of stably expressed internal reference genes is a crucial task of qPCR assay for data analysis. The rationale for reference gene selection is that a lower standard deviation of the cycle of threshold (Cq) among the treatments indicates a more stable expression of the gene. The objective of this study was to select reference genes in human colon cancer cells (COLO 205) treated with cottonseed-derived gossypol and bioactive extracts along with bacterial endotoxin lipopolysaccharides (LPS). SYBR Green qPCR was used to analyze the mRNA levels of a wide range of biomarkers involved in glucose transport, lipid biosynthesis, inflammatory response, and cancer development. qPCR data (10,560 Cq values) were generated from 55 genes analyzed from 64 treatments with triplicate per treatment for each gene. The data showed that B-cell lymphoma 2 (*Bcl2*) mRNA was the most stable among the 55 mRNAs analyzed in the human colon cancer cells. Glyceraldehyde 3 phosphate dehydrogenase (*Gapdh*) and ribosome protein L32 (*Rpl32*) mRNAs were not good qPCR references for the colon cancer cells. These observations were consistent regardless of the treatment comparison between gossypol and LPS, glanded and glandless seed extracts, seed coat and kernel extracts, or treatment for 8 and 24 h. These results suggest that *Bcl2* is a preferable reference gene for qPCR assays in human colon cancer cells treated with cottonseed-derived gossypol and bioactive extracts as well as LPS. The extensive qPCR results firmly support the conclusion that the *Bcl2* gene is stably expressed at the mRNA level in the human colon cancer cells regardless of the treatment, suggesting that *Bcl2* gene expression is not regulated at the mRNA level but at the post-transcriptional level. These results should facilitate studies designated to evaluate bioactivity on gene expression regulation by cottonseed molecules and other natural and synthetic molecules for nutrition and health uses.

## 1. Introduction

**Figure 1:**
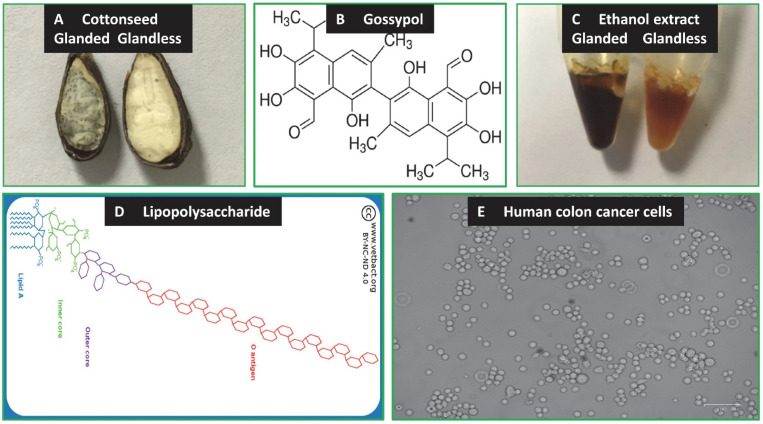
Cottonseed, cottonseed-derived gossypol and polyphenolic extracts, bacteria-derived LPS, and human colon cancer cells. (**A**) Cottonseed (glanded and glandless seed). Glanded seed contains numerous dark-green-colored gossypol glands. (**B**) Cottonseed-derived gossypol (molar mass: 518.56 g/mol). It contains 6 -OH groups and 6 -CH_3_ groups (image was taken from public domain, Gossypol—Wikipedia.) (**C**) Cottonseed-derived ethanol extracts. Cottonseed extracts were isolated by fractionation, defatting, and ethanol extraction from cottonseed coats and kernels of glanded and glandless seeds [1]. (**D**) Bacteria-derived endotoxin LPS. Intact LPS is made up of three structural components (10–20 kDa) [21]: a hydrophobic lipid section, lipid A, which is responsible for the toxic properties of the molecule; a hydrophilic core polysaccharide chain; and a repeating hydrophilic O-antigenic oligosaccharide side chain that is specific to the bacterial serotype. (http://www.vetbact.org/popup/popup.php?id=73, accessed on 1 October 2022). (**E**) Human colon cancer cells used in the study.

The cotton (*Gossypium hirsutum* L.) plant provides economically important fiber and cottonseed but cottonseed only contributes to approximately 20% of the crop value. It is either glanded or glandless depending on its seed with or without gossypol glands (Figure 1A) [1,2]. Cottonseed contains many bioactive molecules including gossypol (Figure 1B), quercetin, gallic acid, 3,4-dihydroxybenzoic acid, flavonoids, cyclopropenoid fatty acids, and peptides [3,4,5,6,7,8,9,10]. Most of these value-added products possess health promotion and disease prevention potentials [6,11,12,13,14,15]. Cottonseed value could be potentially increased by providing high-value bioactive products because plant-derived bioactive materials have been used for disease prevention and treatment since ancient history [16,17,18,19,20].

One of the bioactive materials derived from cottonseed is gossypol, a plant polyphenol with a highly colored yellow pigment found in the leaves, stems, roots, and seeds of cotton plants (Figure 1B) [22]. Gossypol and related compounds are reported to have anticancer activities associated with breast cancer [23,24,25], colon cancer [26,27], pancreatic cancer [28,29], and prostate cancer [30,31]. Gossypol has additional bioactivities such as antiobesity [25], anti-inflammatory [32], and antifungal activities [33]. These new discoveries have generated intensive interest in gossypol and related molecules in the biomedical field.

The other bioactive materials derived from cottonseed are polyphenolic extracts (Figure 1C). Beneficial plant polyphenolic extracts are present in most diets [34]. They regulate gene expression in numerous studies [35,36,37,38,39,40,41]. We recently isolated bioactive ethanol extracts from glanded and glandless cottonseed which were shown to be essentially free of gossypol by HPLC-MS analysis [1]. These bioactive cottonseed extracts also regulate gene expression in mammalian cells [42,43].

The long-term objective of our current research was to explore the potential anti-colon cancer materials from natural sources, especially cottonseed. Colon cancer is one of the deadliest diseases in the world. The lifetime risk of developing colorectal cancer is approximately 4.0% for men and women in 2022 (https://www.cancer.org/cancer/colon-rectal-cancer/about/key-statistics.html, accessed on 1 October 2022). It is urgently needed. access to fully understand the mechanism of developing colon cancer and explore ways to ease the burden of the healthcare crisis. However, there was a lack of molecular evidence for the bioactivity of cottonseed-derived materials in colon cancer cells.

Quantitative real-time-PCR (qPCR) for gene expression analysis is a widely used method for evaluating the bioactivity of natural products. Some questions can be easily answered by qPCR, e.g., the number of isoforms, the levels of gene expression, and the expression patterns of genes regulated by various stimuli. However, the reliability and reproducibility of qPCR results can be affected by many genetic, environmental, and experimental factors because of the high sensitivity [44]. Therefore, one of the critical tasks of qPCR assay design is to select stably expressed internal reference genes for data analysis due to the inherited variations of gene expression among individual organisms, various tissues, different experimental stages and RNA stability, experimental variations such as RNA extraction methods and cDNA preparations, and human errors [45,46]. Carefully selected internal reference mRNAs are used to normalize transcript levels of test genes to more accurately detect the variations [47,48,49,50]. The rationale for qPCR reference gene selection is that the reference gene should be stably expressed without much variation by the experimental treatments. A lower standard deviation of the cycle of threshold (Cq) among the treatments might be an indication of a more stable expression of the gene which could serve as an internal reference. 

The objective of this study was to characterize potential reference genes in human colon cancer cells treated with cottonseed-derived gossypol and bioactive extracts. The well-known bacterial endotoxin lipopolysaccharide (LPS) was selected for comparison with cottonseed materials during the qPCR analysis of gene expression. LPS is a major cell wall component of Gram-negative bacteria (Figure 1D). LPS is widely present in the gut and may be derived from colonial bacteria and/or food contamination. LPS was proposed to have an antitumor effect in several experimental models [51]. One study found that LPS induced TGFβ and HGF production mediated by CD14/TLR-2 in cultured human colon cancer cell lines [52]. Another study showed that LPS promoted NFkB activation in colon cancer cells [53]. A third study demonstrated that LPS promoted the migratory capacity of colon cancer cells with the activation of the SDF-1α/CXCR4 axis and epithelial–mesenchymal transition occurrence [54].

In this study, human colon cancer cells (COLO 205) (Figure 1E) were treated with cottonseed-derived gossypol and ethanol extracts, along with LPS. SYBR Green qPCR was used to analyze the mRNA levels of a wide range of biomarkers involved in glucose transport, lipid biosynthesis, inflammatory response, and cancer development. The data showed that B-cell lymphoma 2 (*Bcl2*) mRNA was the most stable among the 55 mRNAs analyzed in human colon cancer cells. Glyceraldehyde 3 phosphate dehydrogenase (*Gapdh*) and ribosome protein L32 (*Rpl32*) mRNAs, two widely used references in mammalian cells, were not good qPCR references for colon cancer cells. These results suggest that *Bcl2* is a preferable reference gene for qPCR assay of gene regulation by cottonseed and bacterial products in human colon cancer cells. The extensive qPCR results firmly support the conclusion that the *Bcl2* gene is stably expressed at the mRNA level in the human colon cancer cells regardless of the treatment, suggesting that *Bcl2* gene expression is not regulated at the mRNA level but at the post-transcriptional level.

## 2. Results

### 2.1. Cq mean Distribution in Colon Cancer Cells Treated with Gossypol, LPS, and Cottonseed Extracts

**Figure 2:**
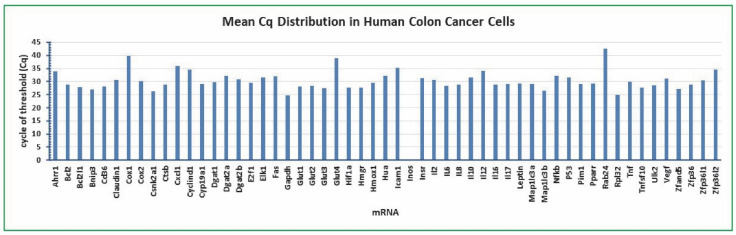
Mean Cq distribution of 55 mRNAs in human colon cancer cells. The cancer cells were treated with multiple concentrations of gossypol (0, 0.1, 0.5, 1, 5, 10, 50, and 100 µg/mL), LPS (0, 5, 10, 20, 50, 100, 500, and 1000 ng/mL) and cottonseed extracts (0, 5, 10, 20, 30, 40, 50, and 100 µg/mL) for 8 h and 24 h. Total mRNAs were extracted from the cells, converted into cDNAs, and the relative abundance was analyzed by SYBR Green qPCR. The Cq values represent the mean of 192 independent samples.

The ideal reference gene should not be expressed at extreme levels. In qPCR technology, cDNA is doubled per cycle of PCR amplification, i.e., one Cq difference equates to a 2-fold difference at the mRNA levels [48]. A lower Cq means a higher mRNA level and vice versa. SYBR Green qPCR assay with the specific primers (Table 1) was used to measure the relative mRNA levels of 55 genes in the cells treated with plant toxin gossypol, bacterial toxin LPS, and cottonseed extracts with 1% DMSO as the control. The qPCR assay showed that *Bcl2* Cq was 28.82 and *Inos* mRNA was undetectable (mean of 192 independent samples) (Figure 2). 

The mean Cq values of 11 mRNAs (mean of 192 independent samples) were at least one Cq less than *Bcl2* Cq including *Bcl2l1* (−1.00), *Bnip3* (−1.75), *Csnk2a1* (−2.43), *Gapdh* (−4.17), *Glut3* (−1.42), *Hif1a* (−1.13), *Hmgr* (−1.21), *Map1lc3b* (−2.17), *Rpl32* (−3.92), *Tnfsf10* (−1.19), and *Zfand5* (−1.67) (Figure 2). The mean Cq values of 24 mRNAs (mean of 192 independent samples) were at least one Cq larger than *Bcl2* Cq including *Ahrr1* (5.15), *Claudin1* (1.92), *Cox1* (10.99), *Cox2* (1.29), *Cxcl1* (7.07), *Cyclind1* (5.71), *Dgat2a* (3.52), *Dgat2b* (2.15), *Elk1* (2.87), *Fas* (3.17), *Glut4* (10.21), *Hua* (3.38), *Icam1* (6.40), *Inos* (undetected), *Insr* (2.59), *Il2* (1.88), *Il10* (2.79), Il12 (5.17), *Nfkb* (3.50), *P53* (2.79), *Rab24* (13.82), *Tnf* (1.23), *Vegf* (2.35), *Zfp36l1* (1.69), and *Zfp36l2* (5.66) (Figure 2).

The above Cq data suggest that the *Bcl2* mRNA level was within the middle range of the tested 55 genes in the human colon cancer cells treated with gossypol, LPS, and cottonseed extracts. The mRNA levels of *Gapdh* and *Rpl32* were the most abundant in the cells with approximately 18- and 15-fold of *Bcl2* mRNA, respectively, whereas *Inos* mRNA was undetectable and those of *Ahrr1*, *Cox1*, *Cxcl1*, *Cyclind1*, *Dgat2a*, *Glut4*, *Hua*, *Icam1*, *Il12*, *Nfkb*, *Rab24*, and *Zfp36l2* mRNAs were minimally detected with less than 10% of *Bcl2* mRNA in the colon cancer cells. These results suggest that the *Bcl2* gene is modestly expressed (i.e., not at an extreme level among the selected biomarkers analyzed) in the human colon cancer cells treated with gossypol, LPS, and cottonseed extracts and that the *Bcl2* gene could serve as a good reference gene for qPCR analysis of gene expression in the human colon cancer cells.

### 2.2. Cq standard Deviation Distribution in Colon Cancer Cells Treated with Gossypol, LPS, and Cottonseed Extracts

**Figure 3:**
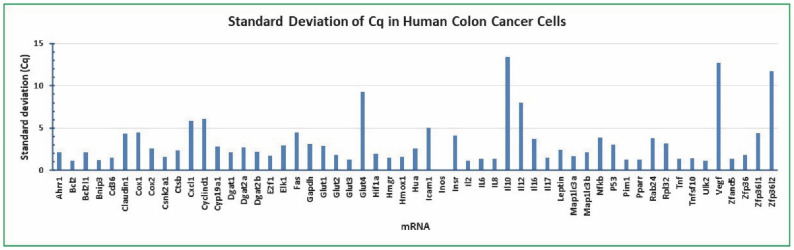
Mean standard deviation distribution of 55 mRNAs in human colon cancer cells. The cancer cells were treated with multiple concentrations of gossypol (0, 0.1, 0.5, 1, 5, 10, 50, and 100 µg/mL), LPS (0, 5, 10, 20, 50, 100, 500, and 1000 ng/mL) and cottonseed extracts (0, 5, 10, 20, 30, 40, 50, and 100 µg/mL) for 8 h and 24 h. Total mRNAs were extracted from the cells, converted into cDNAs, and the relative abundance was analyzed by SYBR Green qPCR. The standard deviations of Cq values represent the mean of 192 independent samples.

The qPCR assay showed that *Bcl2* Cq was the least varied mRNA among the 55 genes analyzed. The standard deviation of Cq for *Bcl2* was 1.09 (mean of 192 independent samples) (Figure 3). The standard deviations of Cq for *Gapdh* and *Rpl32* were 3.12 and 3.16 (mean of 192 independent samples), respectively (Figure 3). All of the other mRNAs had larger standard deviations of Cq than *Bcl2* (Figure 3). The mRNAs with closest standard deviations of *Bcl2* Cq (less than 1.50) included *Bnip3* (1.21), *Glut3* (1.30), *Hmgr* (1.49), *Il2* (1.12), *Il6* (1.37), *Il8* (1.32), *Il17* (1.48), *Pim1* (1.26), *Pparr* (1.32), *Tnf* (1.39), *Tnfsf10* (1.40), *Ulk2* (1.16), and *Zfand5* (1.34) (Figure 3). These results suggest that *Bcl2* is the most stably expressed gene and therefore, could serve as a reliable reference for qPCR analysis of gene expression in human colon cancer cells treated with gossypol, LPS, and cottonseed extracts, regardless of the treatments.

### 2.3. Variation of Gene Expression between Plant Toxin Gossypol and Bacterial Toxin LPS Treatment

**Figure 4:**
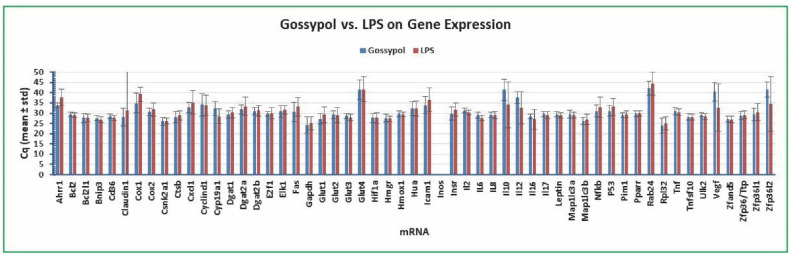
Variation of gene expression between gossypol and LPS treatment. The cancer cells were treated with multiple concentrations of gossypol (0, 0.1, 0.5, 1, 5, 10, 50, and 100 µg/mL) and LPS (0, 5, 10, 20, 50, 100, 500, and 1000 ng/mL) for 8 h. Total mRNAs were extracted from the cells, converted into cDNAs, and the relative abundance was analyzed by SYBR Green qPCR. The Cq values represented the mean and standard deviation of 24 independent samples.

To confirm the above conclusion that *Bcl2* was a preferable reference gene for qPCR analysis of gene expression in the human colon cancer cells treated with gossypol, LPS, and cottonseed extracts, we selected subsets of data for comparison. We first compared the variation of gene expression in the human colon cancer cells under the treatment with plant toxin gossypol and bacterial toxin LPS. As shown in Figure 4, *Bcl2* Cq was the least varied among the 55 targets with a standard deviation of 1.08 and 1.16 for gossypol and LPS treatment (mean of 24 independent samples), respectively. The standard deviations of *Gapdh* Cq were 4.01 (gossypol treatment) and 3.15 (LPS treatment) and those of *Rpl32* Cq were 3.68 (gossypol treatment) and 3.10 (LPS treatment) (mean of 24 independent samples) (Figure 4). All of the other mRNAs had larger standard deviations of Cq than *Bcl2* Cq (Figure 4). The mRNA levels of genes regulated by gossypol that were at least 2-fold of those regulated by LPS could be interpreted as gossypol regulation of gene expression being significantly higher than LPS. There were 14 genes with more abundantly expressed mRNA levels under gossypol treatment than LPS treatment including *Ahrr1*, *Claudin1*, *Cox1*, *Cox2*, *Cxcl1*, *Dgat2a*, *Fas*, *Glut1*, *Icam1*, *Insr*, *Nfkb*, *P53*, *Rab24*, and *Zfp36l1* (Figure 4). Similarly, the mRNA levels of genes regulated by gossypol less than 0.5-fold of those regulated by LPS could be interpreted as their expression being regulated by gossypol significantly less than the LPS effect. There were 7 genes with less abundantly expressed mRNA levels under gossypol treatment than under LPS treatment including *Cyp19a1*, *Il6*, *Il10*, *Il12*, *Il16*, *Vegf*, and *Zfp36l2* (Figure 4). The above results of modest expression of the *Bcl2* gene with minimal variation agree with the conclusion that *Bcl2* is a suitable reference gene for qPCR analysis of gene expression in human colon cancer cells regardless of treatments with gossypol or LPS.

### 2.4. Variation of Gene Expression between Glanded and Glandless Cottonseed Extract Treatment

**Figure 5:**
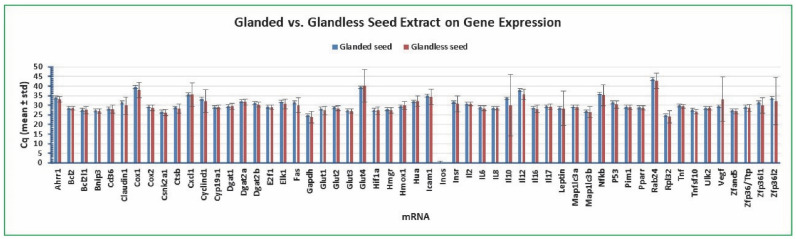
Variation of gene expression between glanded and glandless cottonseed extract treatment. The cancer cells were treated with multiple concentrations of cottonseed extracts (0, 5, 10, 20, 30, 40, 50, and 100 µg/mL) for 8 h. Total mRNAs were extracted from the cells, converted into cDNAs, and the relative abundance was analyzed by SYBR Green qPCR. The Cq values represented the mean and standard deviation of 48 independent samples.

We further compared the variation of gene expression in the human colon cancer cells under the treatment with glanded and glandless cottonseed extracts. Again, *Bcl2* Cq was the least varied among the 55 targets with a standard deviation of 1.07 and 0.98 for glanded seed extract and glandless seed extract (mean of 48 independent samples), respectively (Figure 5). The standard deviations of *Gapdh* Cq were 3.34 (glanded seed extract) and 3.51 (glandless seed extract) and those of *Rpl32* Cq were 3.22 (glanded seed extract) and 3.43 (glandless seed extract) (mean of 48 independent samples) (Figure 5). All of the other mRNAs had larger standard deviations of Cq than *Bcl2* Cq (Figure 5). There were only two genes more abundantly expressed under glanded seed extract treatment than glandless seed extract treatment including *Vegf* and *Zfp36l1* (Figure 5). There were 15 genes with lower mRNA levels under glanded seed extract treatment than under glandless seed extract treatment including *Claudin1*, *Cox1*, *Cyclind1*, *Elk1*, *Fas*, *Gapdh*, *Glut1*, *Insr*, *Il10*, *Il12*, *Nfkb*, *P53*, *Rab24*, *Zfp36l1*, and *Zfp36l2* (Figure 5). These results support the conclusion that *Bcl2* is a suitable reference gene for qPCR analysis of gene expression in human colon cancer cells regardless of treatment with glanded or glandless cottonseed extracts.

### 2.5. Variation of Gene Expression between Cottonseed Coat and Kernel Extract Treatment

**Figure 6:**
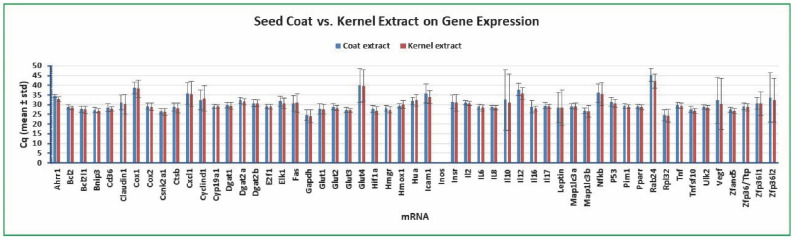
Variation of gene expression between cottonseed coat and kernel extract treatment. The cancer cells were treated with multiple concentrations of cottonseed extracts (0, 5, 10, 20, 30, 40, 50, and 100 µg/mL) for 8 h. Total mRNAs were extracted from the cells, converted into cDNAs, and the relative abundance was analyzed by SYBR Green qPCR. The Cq values represented the mean and standard deviation of 48 independent samples.

We also compared the variation of gene expression under the treatment with seed coat and kernel extracts in the human colon cancer cells. As shown in Figure 6, *Bcl2* Cq was the least varied mRNA among the 55 targets with a standard deviation of 1.06 and 0.96 for coat extract and kernel extract (mean of 48 independent samples), respectively. The standard deviations of *Gapdh* Cq were 3.05 (coat extract) and 3.83 (kernel extract) and those of *Rpl32* Cq were 3.18 (coat extract) and 3.49 (kernel extract) (mean of 48 independent samples), respectively (Figure 6). All of the other mRNAs had larger standard deviations of Cq than *Bcl2* Cq (Figure 6). There were none of the genes with more abundantly expressed mRNA levels under the coat extract treatment than the kernel extract treatment (Figure 6). There were 13 genes with less abundantly expressed mRNA levels under coat extract treatment than kernel extract treatment including *Ahrr1*, *Cox1*, *Elk1*, *Gapdh*, *Icam1*, *Insr*, *Il10*, *Il12*, *Nfkb*, *Rab24*, *Vegf*, *Zfp36l1*, and *Zfp36l2* (Figure 6). The conclusion that *Bcl2* is a suitable reference gene for qPCR analysis of gene expression in human colon cancer cells is confirmed with this comparison regardless of the cells treated with seed coat or kernel extract.

### 2.6. Variation of Gene Expression between Cottonseed Extract Treatment Time

**Figure 7:**
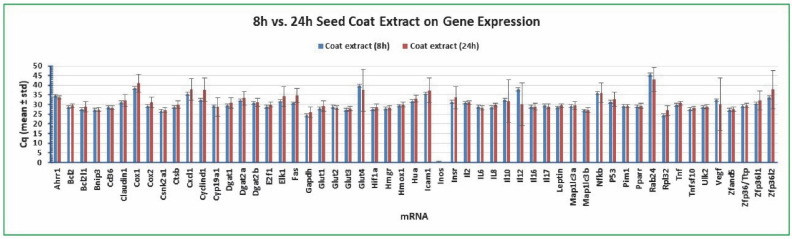
Variation of gene expression between 8 and 24 h treatment of coat extract of cottonseed. The cancer cells were treated with multiple concentrations of cottonseed extracts (0, 5, 10, 20, 30, 40, 50, and 100 µg/mL) for 8 h. Total mRNAs were extracted from the cells, converted into cDNAs, and the relative abundance was analyzed by SYBR Green qPCR. The Cq values represented the mean and standard deviation of 48 independent samples.

In addition, we compared the variation of gene expression in the human colon cancer cells between 8 and 24 h treatment with seed coat extract. As shown in Figure 7, *Bcl2* Cq was the least varied mRNA among the 55 targets with a standard deviation of 1.06 and 0.95 for 8 h and 24 h (mean of 48 independent samples), respectively. The standard deviations of *Gapdh* Cq were 3.05 (8 h) and 2.90 (24 h) and those of *Rpl32* Cq were 3.18 (8 h) and 2.93 (24 h) (mean of 48 independent samples), respectively (Figure 7). All of the other mRNAs had larger standard deviations of Cq than *Bcl2* Cq (Figure 7). There were 21 genes with more abundantly expressed mRNA levels under 8 h treatment than 24 h treatment including *Bcl2l1*, *Claudin1*, *Cox1*, *Cox2*, *Cstb*, *Cxcl1*, *Cylind1*, *Dgat1*, *Elk1*, *Fas*, *Gapdh*, *Glut1*, *Hua*, *Icam1*, *Insr*, *Il8*, *Nfkb*, *P53*, *Rpl32*, *Zfp36l1*, and *Zfp36l2* (Figure 7). There were 5 genes with less abundantly expressed mRNA levels under 8 h treatment than 24 h treatment including *Ahrr1*, *Glut4*, *Il12*, *Rab24*, and *Vegf* (Figure 7). These results of gene expression variation in the human colon cancer cells under 8 or 24 h treatment with seed coat extract also support the conclusion that *Bcl2* but not *Gapdh* or *Rpl32* or any other gene is a preferable reference gene for qPCR analysis 

### 2.7. Variation of Gene Expression between DMSO Control and Various Treatments

**Figure 8:**
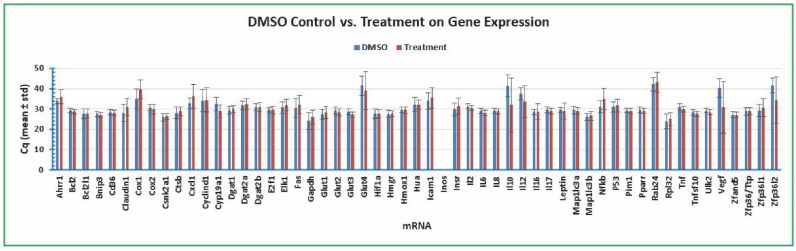
Variation of gene expression between DMSO control and treatment. The cancer cells were treated with 1% DMSO and multiple concentrations of gossypol (0.1, 0.5, 1, 5, 10, 50, and 100 µg/mL), LPS (5, 10, 20, 50, 100, 500, and 1000 ng/mL) and cottonseed extracts (5, 10, 20, 30, 40, 50, and 100 µg/mL) for 8 h and 24 h. Total mRNAs were extracted from the cells, converted into cDNAs, and the relative abundance was analyzed by SYBR Green qPCR. The Cq values represented the mean and standard deviation of 24 independent samples.

Finally, we compared the variation of gene expression in the human colon cancer cells under the 1% DMSO control and various treatments with plant toxin gossypol, bacterial toxin LPS, and cottonseed-derived ethanol extracts. Figure 8 confirms that *Bcl2* was the most stable gene among the 55 targets with standard deviations of 1.08 and 1.08 for DMSO control (mean of 24 independent samples) and treatment (mean of 168 independent samples). The standard deviations of *Gapdh* Cq were 4.01 (DMSO control) and 3.57 (treatment) and those of *Rpl32* Cq were 3.68 (DMSO control) and 3.07 (treatment) (Figure 8). All of the other mRNAs had larger standard deviations of Cq than *Bcl2* Cq (Figure 8). These qPCR data analyses further support the above conclusion that *Bcl2* gene is stably expressed at the mRNA level in human colon cancer cells regardless of the treatment.

## 3. Discussion

Cottonseed is a low-value commodity and contributes to approximately 20% of the cotton value. Cottonseed value could be increased by serving as a cheap source of high-value bioactive materials for improving nutrition and preventing diseases. However, molecular evidence to support cottonseed bioactivity in mammalian cells is not substantial. 

qPCR is widely used to evaluate the bioactivity of natural products at the gene expression level. However, many factors affect the calculation of gene expression data due to the inherited variations of gene expression among individual organisms, various tissues, different experimental stages, and RNA stability. It is also affected by experimental variations such as RNA extraction methods and cDNA preparations, and human errors [44,46]. Therefore, the selection of stably expressed internal reference genes is a critical task for qPCR assay design for normalizing transcript levels of test genes during the post-qPCR data analysis [48,49].

The hallmark of qPCR reference gene selection is that a reference gene should be stably expressed without much variation by experimental conditions and ideally its expression level is like those of the target genes [45,97]. A lower standard deviation of Cq among the treatments indicates a more stable expression of the gene which could serve as a better internal reference. In this study, we used the qPCR method to screen reference genes from 55 genes involved in glucose transport, lipid biosynthesis, inflammatory response, and cancer development using human colon cancer cells treated with multiple concentrations of plant toxin gossypol, bacterial toxin LPS, and bioactive cottonseed extracts. Our results consistently showed that *Bcl2* is a very stably expressed gene with minimal variation and expressed at adequate mRNA levels similar to most of the other gene targets. On the other hand, two widely used reference genes (*Gapdh* or *Rpl32*) were the most abundantly expressed genes with much larger variations among the treatments. These expression differences among them make *Bcl2* rather than *Gapdh* or *Rpl32* a preferable qPCR reference for colon cancer cells. These conclusions were confirmed by various comparisons between gossypol and LPS, glanded and glandless seed extracts, seed coat and kernel extracts, or treatment for 8 and 24 h. A previous study analyzed the effect of gossypol on gene expression in human colon cancer cells, using *Bcl2* as the reference gene [26]. The current study is technically oriented rather than looking at the regulation of specific genes with specific agents. This study has analyzed much larger data sets with a broader new view. The comparisons among the treatments are also much more comprehensive. All of the data support the conclusion that *Bcl2* is a preferable reference gene for qPCR assay of gene expression in human colon cancer cells.

Our current results firmly support the conclusion that *Bcl2* is a preferable reference gene for qPCR assay of gene expression in human colon cancer cells treated with cottonseed molecules and bacterial endotoxin LPS. This conclusion is drawn from extensive qPCR data with 55 genes analyzed and 192 individual samples (64 treatments with triplicate for each treatment) for each gene for a total of 10,560 Cq values. Since our study used regulatory molecules ranging from plant toxin gossypol to bacterial endotoxin LPS as well as bioactive cottonseed mixtures, it is expected that the technical advance should be applicable to evaluating a wide range of biomolecules’ modulation of gene expression in the human colon cancer cells. However, further research is required if *Bcl2* is a preferable reference gene for qPCR assay of gene expression in other cell lines since responses of stimuli on gene expression are widely different in different cell types. 

Our extensive qPCR results support the conclusion that the *Bcl2* gene is stably expressed at the mRNA level in human colon cancer cells regardless of the treatment with plant toxin gossypol, bacterial endotoxin LPS, or cottonseed-derived bioactive extracts. However, a number of previous studies reported the regulation of *Bcl2* gene expression in other testing systems, even though the findings in those reports were mostly generated by less sensitive methods such as immunoblotting, immunostaining, and end-point PCR techniques. For example, Western blotting showed that *Bcl2* protein levels in colorectal cancer HCT-116 cells were reduced to less than half of the control by 5 days of treatment with 25–100 µg/mL of 3,6-anhydro-L-galactose derived from red seaweed agarose [98]. Similarly, fermented Pu-erh tea (Xiaguan bowl tea [X]) decreased *Bcl2* gene expression in HT-29 colon cancer cells [99]. In situ hybridization and immunostaining showed that tea polyphenol treatment significantly reduced the percentage of *Bcl2* expressing cells and reduced the level of *Bcl2* mRNA and protein in the *Bcl2* positive cells in lung preneoplastic lesions of Sprague Dawley rats [100]. Curcumin (diferuloylmethane), the yellow pigment in turmeric (*Curcuma longa*), at 25 µM for 4 h suppressed NFκB-regulated gene products (*Bcl2*, *BclxL*, *Cox2*, cyclin D1, and inhibitor of apoptosis protein-2) in HCT-116 cells [101]. Western blotting showed that curcumin treatment at 20 µM for 24 h but not 6 h or 12 h inhibited the expression of *Bcl2* by about 50% in COLO 205 cells [102]. Regular end-point PCR showed that methanol extract from the plant *Drimia calcarata* significantly downregulated *Bcl2* gene expression in both Caco-2 and HT-29 cells [103]. Finally, Western blot and qPCR showed that treatment with berberine (40 μM), a natural isoquinoline alkaloid derived from *Berberis* genus plants, decreased the expression of *Bcl2* protein but not mRNA in human colorectal cancer cell lines HT-29 and HCT-116 [104]. Taken together, all of the above results strongly suggest that *Bcl2* gene expression is not regulated at the mRNA level but at the post-transcriptional level.

In conclusion, this study identified *Bcl2* as a preferable reference gene for qPCR assays in human colon cancer cells treated with cottonseed-derived gossypol and bioactive extracts as well as LPS. Our extensive qPCR results firmly support the conclusion that the *Bcl2* gene is stably expressed at the mRNA level in human colon cancer cells regardless of the treatment with plant toxin gossypol, bacterial endotoxin LPS, or cottonseed-derived bioactive extracts, suggesting that *Bcl2* gene expression is not regulated at the mRNA level but at the post-transcriptional level. These results should facilitate studies designated to evaluate the bioactivity of cottonseed molecules and other natural and synthetic molecules for nutrition and health uses.

## 4. Materials and Methods

### 4.1. Colon Cancer Cell Line

American Type Culture Collection (Manassas, VA, USA) provided the human colon cancer cell line (COLO 205-ATCC CCL-222). For long-term storage, the cells were kept under liquid nitrogen vapor in a cryogenic storage vessel (Thermo Fisher Scientific, Waltham, MA, USA). During the experiment, the cells were maintained at 37 °C in a humidified incubator with 5% CO_2_ in RPMI-1640 medium (Gibco, Life Technologies, Carlsbad, CA, USA) supplemented with 10% (v:v) fetal bovine serum, 0.1 million units/L penicillin, 100 mg/L streptomycin, and 2 mmol/L L-glutamine. 

### 4.2. Gossypol, LPS, and Cottonseed Extracts

Gossypol (molar mass: 518.56 g/mol) was purified from cottonseed by HPLC (Sigma, St. Louis, MO, USA). LPS was extracted from *E. coli* serotype K235 and purified by gel filtration (Sigma, St. Louis, MO, USA). Cottonseed extracts were isolated by fractionation, defatting, and ethanol extraction from cottonseed coats and kernels of glanded and glandless seeds [1]. Briefly, the cottonseed coat or kernel was ground into a fine powder and homogenized. The kernel fraction was defatted with chloroform and hexane. The coat fraction was treated with acetic acid followed by autoclave and centrifugation. The defatted materials were extracted with ethanol followed by evaporation to remove acetic acid and ethanol. Ethanol extracts were reconstituted in 100% DMSO (100 mg/mL) (Sigma, St. Louis, MO, USA) and analyzed by HPLC-MS. The ethanol extracts contained trace amounts of gossypol (0.82 ng gossypol/mg extract in glanded seed coat, 0.03 ng gossypol/mg extract in glanded seed kernel, 0.37 ng gossypol/mg extract in glandless seed coat, and 0 ng gossypol/mg extract in glandless seed kernel) [1]. Gossypol, LPS, and cottonseed extract stocks were prepared in 100% DMSO and diluted before use.

### 4.3. Reagents and Equipment

Tissue culture reagents (RPMI-1640, fetal bovine serum, penicillin, streptomycin, and L-glutamine) were from Gibco BRL. The tissue culture incubator (water jacket CO_2_ incubator, Forma Series II, Model 3100 Series) was from Thermo Fisher. The tissue culture workstation (Logic+ A2 hood) was from Labconco (Kansas City, MO, USA). Tissue culture plastic (flasks, plates, cell scraper) was from CytoOne (USA Scientific, Ocala, FL, USA). Cell counting reagent (trypsin blue dye), slides (dual chamber), counter (TC20 Automatic Cell Counter), and microscope (Zoe Florescent Cell Imager) were from Bio-Rad (Hercules, CA, USA). The microplate spectrophotometer (Epoch) was from BioTek Instruments (Winooski, VT, USA).

### 4.4. Cell Culture and Chemical Treatment

Cancer cells were dissociated from the T-75 flask with 0.25% (*w/v*) trypsin−0.53 mM EDTA solution, stained with an equal volume of 0.4% trypsin blue dye before counting the number of live cells with a TC20 Automatic Cell Counter. Cancer cells (0.5 mL) from trypsin-dissociated flasks were subcultured at approximately 1 × 10^5^ cells/mL density in 24-well tissue culture plates. The cancer cells were routinely observed under a Zoe Florescent Cell Imager before and during the treatment. Cancer cells in 24-well plates (triplicate for every treatment) were treated with gossypol (0, 0.1, 0.5, 1, 5, 10, 50, and 100 µg/mL), LPS (0, 5, 10, 20, 50, 100, 500, and 1000 ng/mL), or ethanol extracts (0, 5, 10, 20, 30, 40, 50, and 100 µg/mL) for 8 and 24 h. The experimental control “0” treatment corresponded to 1% DMSO present in all of the culture mediums. Gossypol concentrations were in the range of previously published concentrations for gossypol (up to 100 µM) [23,25,105], (-) gossypol (up to 100 µM) [106], apogossypolone (up to 40 µM) [107], and gossypol derivatives (IC_50_ concentrations of 6–28 µM) [108]. 

### 4.5. Real-Time qPCR Primers and Probes

The selection of 55 genes for qPCR analysis was based on the literature showing those gene expressions regulated by TTP, gossypol, or cinnamon extract in cancer cells and macrophages (relevant references are listed in the right column in Table 1). RNA sequences were obtained from the National Center for Biotechnology Information (NCBI)’s non-redundant protein sequence databases (http://blast.ncbi.nlm.nih.gov/Blast.cgi). The qPCR primers were designed with Primer Express 3.0 software (Applied Biosystems, Foster City, CA, USA) using the default parameter values including amplicon length (50 to 150 bases for optimum PCR efficiency), optimal primer length (20 bases), Tm (58 °C to 60 °C), % GC (30% to 80%), 3′ end (the last five nucleotides at the 3′ end contain no more than two G + C residues), and repeating oligonucleotides (void runs of identical nucleotides; if repeats are present, there must be fewer than four consecutive G residues). The primers were synthesized by Biosearch Technologies, Inc. (Navato, CA, USA). The names of mRNAs and their nucleotide sequences (5′ to 3′) of the forward primers and reverse primers, and corresponding references are described in Table 1.

### 4.6. RNA Isolation and cDNA Synthesis

RNA isolation and cDNA synthesis were essentially as described [13]. Human colon cancer cells in 24-well plates treated with various concentrations of gossypol, LPS, or cottonseed extracts for 8 h (triplicate in every concentration). The dishes were washed twice with 1 mL 0.9% NaCl and lysed directly with 1 mL of TRI_ZOL_ reagent (Invitrogen, Carlsbad, CA, USA). RNA was isolated according to the manufacturer’s instructions without DNase treatment and stored in a −80 °C freezer. RNA concentrations were quantified with an Implen NanoPhotometer (Munchen, Germany). The mean and standard deviations of A260/A280 for the 192 independent RNA samples was 1.73 ± 0.17, indicating some contaminations of the RNA samples with protein, phenol, or other contaminants that have an absorbance close to 280 nm. RNA quality was evaluated by electrophoretic gel and electropherogram analyses. In our typical analysis, RNA isolated from mammalian cells with the Trizol reagent without DNase treatment resulted in high-quality RNA as evidenced by sharp 28S and 18S rRNA bands on electrophoretic gel and sharp peaks on electropherogram [109]. The total RNA was used to synthesize cDNAs using SuperScript II reverse transcriptase at 42 °C for 50 min. The cDNA synthesis mixture (20 μL) contained 5 μg total RNA, 2.4 μg oligo(dT)_12–18_ primer, 0.1 μg random primers, 500 μM dNTPs, 10 mM DTT, 40 u RNaseOUT and 200 u SuperScript II reverse transcriptase in 1X first-strand synthesis buffer (Life Technologies, Carlsbad, CA, USA). The cDNA was stored in a −80 °C freezer and diluted with water to 1 ng/µL before qPCR analyses.

### 4.7. Quantitative Real-Time PCR Analysis

The qPCR assays followed the MIQE guidelines: minimum information for publication of quantitative real-time PCR experiments [46]. The qPCR assays were described in detail previously [26]. The qPCR efficiency was performed with variable amounts of template cDNA concentrations (0, 0.05, 0.5, 2.5, 5, 12.5, and 25 ng) essentially as described [109]. The correlation coefficiencies between Cq and cDNA concentration were over 0.99 for the BCL2 gene and tested genes. SYBR Green qPCR reaction mixture (12.5 μL) contained 5 ng of total RNA-derived cDNA, 200 nM each of the forward primer and reverse primer, and 1× iQ SYBR Green Supermix (Bio-Rad Laboratories, Hercules, CA, USA). The reactions in 96-well clear plates sealed by adhesives were performed with CFX96 real-time system-C1000 Thermal Cycler (Bio-Rad Laboratories, Hercules, CA, USA). The thermal cycle conditions were as follows: 3 min at 95 °C, followed by 40 cycles at 95 °C for 10 s, 65 °C for 30 s and 72 °C for 30 s. The specificity of qPCR products was evaluated by melt curve analysis and 3% agarose gel electrophoresis as described [109]. Primer pair specificity for cDNA was evident from the analysis of qPCR products with sharp peaks on the melt curve and a single band on the electrophoretic gel.

### 4.8. Data Analysis and Statistics

qPCR data (10,560 Cq values) were generated from 55 genes analyzed and 64 treatments with triplicate per treatment for each gene. The data in the figures and tables represent the mean and standard deviation of 24–192 independent samples. 

## Figures and Tables

**Table 1:** Human mRNA targets analyzed by qPCR; these genes are regulated by TTP, plant toxin gossypol, and plant nutrient cinnamon extract as indicated in the references.

ID	mRNA	Name	Forward Primer (5' to 3')	Reverse Primer (5' to 3')	Regulator [Reference]
H1	Ahrr1	Aryl hydrocarbon receptor repressor	AGGCTGCTGTTGGAGTCTCTTAA	CGATCGTTGCTGATGCATAAA	TTP [55]
H2	Bcl2	B-cell lymphoma 2	CAGCATGCGGCCTCTGTT	GGGCCAAACTGAGCAGAGTCT	Gossypol [56]
H3	Bcl2l1	B-cell lymphoma 2 like 1	GTGCGTGGAAAGCGTAGACA	ATTCAGGTAAGTGGCCATCCAA	TTP [57]
H4	Bnip3	BCL2 protein-interacting protein 3	GTCAAGTCGGCCGGAAAATA	TGCGCTTCGGGTGTTTAAAG	Gossypol [27]
H5	Cd36	Cluster of differentiation 36/fatty acid translocase	CTCTTTCCTGCAGCCCAATG	TTGTCAGCCTCTGTTCCAACTG	TTP [58]
H6	Claudin1	Maintain tissue integrity and water retention	GACAAAGTGAAGAAGGCCCGTAT	CAAGACCTGCCACGATGAAA	TTP [59]
H7	Cox1	Cyclooxygenase 1	CGCCCACGCCAGTGA	AGGCCGAAGCGGACACA	TTP [60]
H8	Cox2	Cyclooxygenase 2	CGATTGTACCCGGACAGGAT	TTGGAGTGGGTTTCAGAAATAATTT	TTP [61]
H9	Csnk2a1	Casein kinase 2 alpha 1	AGCGATGGGAACGCTTTG	AAGGCCTCAGGGCTGACAA	TTP [62]
H10	Ctsb	Cathepsin B	GACTTGTAGCTGCTGTCTCTCTTTGT	CAAGAGTCGCAAGAACATGCA	TTP [63]
H11	Cxcl1	Chemokine (C-X-C motif) ligand 1	GCCCAAACCGAAGTCATAGC	TGCAGGATTGAGGCAAGCT	TTP [64]
H12	Cyclind1	Cyclin D1	ACACGCGCAGACCTTCGT	CCATGGAGGGCGGATTG	Gossypol [65]
H13	Cyp19a1	Cytochrome P450 family 19 subfamily A member 1	GACATTGCAAGGACAGTGTGTTG	AGTCTCATCTGGGTGCAAGGA	Gossypol [66]
H14	Dgat1	Diacylglycerol O-acyltransferase 1	ACCTCATCTGGCTCATCTTCTTCTA	CCCGGTCTCCAAACTGCAT	Cinnamon [40,67]
H15	Dgat2a	Diacylglycerol O-acyltransferase 2a	CCCAGGCATACGGCCTTA	CAACACAGGCATTCGGAAGTT	Cinnamon [40,68]
H16	Dgat2b	Diacylglycerol O-acyltransferase 2b	ACTCTGGCCCTTCTCTGTTTTTTA	TCCACCTTGGTTGGGTGTGT	Cinnamon [40,68]
H17	E2f1	E2F transcription factor 1	CGGCGCATCTATGACATCAC	CAGCCACTGGATGTGGTTCTT	TTP [69]
H18	Elk1	ETS transcription factor	CTCCTCCGCATCCCTCTTTAA	AGCGTCACAGATGGGTCCAT	TTP [70]
H19	Fas	Fas cell surface death receptor	GAACTCCTTGGCGGAAGAGA	AGGACCCCGTGGAATGTCA	Gossypol [71]
H20	Gapdh	Glyceraldehyde-3-phosphate dehydrogenase	GGGTGTGAACCATGAGAAGTATGA	GGTGCAGGAGGCATTGCT	[72]
H21	Glut1	Glucose transporter 1	TGCTCATGGGCTTCTCGAA	AAGCGGCCCAGGATCAG	Cinnamon [39]
H22	Glut2	Glucose transporter 2	GCATTTTTCAGACGGCTGGTA	GCGCCAACTCCAATGGTT	Cinnamon [39]
H23	Glut3	Glucose transporter 3	GAGGATATCACACGGGCCTTT	CCATGACGCCGTCCTTTC	Cinnamon [39]
H24	Glut4	Glucose transporter 4	CGTGGGCGGCATGATT	CCAGCATGGCCCTTTTCC	Cinnamon [39]
H25	Hif1a	Hypoxia inducible factor 1 subunit alpha	GGTGGATATGTCTGGGTTGAAAC	ATGCACTGTGGTTGAGAATTCTTG	TTP [73]
H26	Hmgr	3-Hydroxy-3-methylglutaryl-CoA reductase	AAGTGAAAGCCTGGCTCGAA	CTAGTGCTGTCAAATGCCTCCTT	[74]
H27	Hmox1	Heme oxygenase 1	CTTCTCCGATGGGTCCTTACACT	TCACATGGCATAAAGCCCTACA	TTP [75]
H28	Hua	Human antigen a	GATCCTCTGGCAGATGTTTGG	CGCGGATCACTTTCACATTG	Gossypol [41]
H29	Icam1	Intercellular adhesion molecule 1/CD54	GGAGCTTCGTGTCCTGTATGG	TTTCTGGCCACGTCCAGTTT	[76]
H30	Inos	Inducible nitric oxide synthase	AGATCCGGTTCACAGTCTTGGT	GCCATGACCTTCCGCATTAG	[77]
H31	Insr	Insulin receptor	CAACGGGCAGTTTGTCGAA	TGGTCGGGCAAACTTTCTG	[38]
H32	Il2	Interleukin 2	TATGCAGATGAGACAGCAACCAT	TTGAGATGATGCTTTGACAAAAGG	TTP [78]
H33	IL6	Interleukin 6	CCCACACAGACAGCCACTCA	CCGTCGAGGATGTACCGAAT	TTP [79]
H34	IL8	Interleukin 8	CCATCTCACTGTGTGTAAACATGACTT	ATCAGGAAGGCTGCCAAGAG	TTP [80]
H35	Il10	Interleukin 10	GCCGTGGAGCAGGTGAAG	TGGCTTTGTAGATGCCTTTCTCT	TTP [81]
H36	Il12	Interleukin 12	TGCCTTCACCACTCCCAAA	TGTCTGGCCTTCTGGAGCAT	TTP [82]
H37	Il16	Interleukin 16	CAGGGCCTCACACGGTTT	GACAATCGTGACAGGTCCATCA	TTP [83]
H38	Il17	Interleukin 17	CCCAAAAGGTCCTCAGATTACTACA	TCATTGCGGTGGAGATTCC	TTP [84]
H39	Leptin	Body fat and obesity hormone	AGGGAGACCGAGCGCTTT	CACATCCCTCACCTCCTTCAAA	[85]
H40	Map1lc3a	Microtubule-associated proteins 1 light chain 3A	GTGAACCAGCACAGCATGGT	CCTCGTCTTTCTCCTGCTCGTA	[86]
H41	Map1lc3b	Microtubule-associated proteins 1 light chain 3B	AGGCGCTTACAGCTCAATGC	ACCATGCTGTGTCCGTTCAC	[86]
H42	Nfkb	Nuclear factor kappa B	GGTGCCTCTAGTGAAAAGAACAAGA	GCTGGTCCCACATAGTTGCA	[87]
H43	P53	Tumor suppressor	CTTGCAATAGGTGTGCGTCAGA	GGAGCCCCGGGACAAA	Gossypol [88]
H44	Pim1	Proto-oncogene serine/threonine-protein kinase	TGCTCCACCGCGACATC	TGAGCTCGCCGCGATT	TTP [89]
H45	Pparr	Peroxisome proliferator-activated receptor γ	GAACGACCAAGTAACTCTCCTCAAA	CAAGGAGGCCAGCATTGTGT	Gossypol [90]
H46	Rab24	Ras-related oncogene 24	TCGGTCGGAGACGCACTT	TGGCCTCATAGCGCTCAGA	[91]
H47	Rpl32	Ribosomal protein L32 (60S ribosomal unit)	CCTCCAAGAACCGCAAAGC	GGTGACTCTGATGGCCAGTTG	[92]
H48	Tnf	Tumor necrosis factor	GGAGAAGGGTGACCGACTCA	CAGACTCGGCAAAGTCGAGAT	TTP [79]
H49	Tnfsf10	Tumor necrosis factor superfamily, member 10	GCTCTGGGCCGCAAAAT	AGGAATGAATGCCCACTCCTT	Gossypol [93]
H50	Ulk2	Unc-51 like autophagy activating kinase 2	ACAGCTCCTTTCAAAATCCCTAAA	AGGCCCATGACGAGTAACCA	[94]
H51	Vegf	Vascular endothelial growth factor	CCCACTGAGGAGTCCAACATC	GGCCTTGGTGAGGTTTGATC	TTP [95]
H52	Zfand5	Zinc finger AN1-type containing 5	AGGGTTTGACTGCCGATGTG	ACTGGATTCTCTTTTCTGATTTTTGC	TTP [96]
H53	Zfp36/Ttp	Zinc finger protein 36/Tristetraprolin	GGCGACTCCCCATCTTCAA	GACCGGGCAGTCACTTTGTC	TTP [38]
H54	Zfp36l1	Zinc finger protein 36 like 1	TCTGCCACCATCTTCGACTTG	TGGGAGCACTATAGTTGAGCATCT	TTP [38]
H55	Zfp36l2	Zinc finger protein 36 like 2	CCTTTCATACCATCGGCTTCTG	TCGTCCGCGTTGTGGAT	TTP [38]

## Data Availability

The datasets generated during the current study are available in the NIH Gene Expression Omnibus (GEO) Database, accession number GSE200980, GSE203027, GSE204818, and GSE204820.

## Sample Availability

Samples of the compounds are available from the authors.

## References

[1] Cao H., Sethumadhavan K., Bland J.M. (2018). Isolation of cottonseed extracts that affect human cancer cell growth. Sci. Rep..

[2] Pons W.A., Hoffpauir C.L., Hopper T.H. (1953). Gossypol in Cottonseed, Influence of Variety of Cottonseed and Environment. J. Agric. Food Chem..

[3] Cao H., Sethumadhavan K. Cottonseed bioactive compounds and peptides. Proceedings of the 2020 Beltwide Cotton Conferences.

[4] Piccinelli A.L., Veneziano A., Passi S., Simone F.D., Rastrelli L. (2007). Flavonol glycosides from whole cottonseed by-product. Food Chem..

[5] Wang X., Howell C.P., Chen F., Yin J., Jiang Y. (2009). Gossypol—A polyphenolic compound from cotton plant. Adv. Food Nutr. Res..

[6] He Z., Zhang D., Olanya O.M. (2020). Antioxidant activities of the water-soluble fractions of glandless and glanded cottonseed protein. Food Chem..

[7] He Z., Nam S., Zhang H., Olanya O.M. (2022). Chemical Composition and Thermogravimetric Behaviors of Glanded and Glandless Cottonseed Kernels. Molecules.

[8] Song W., Kong X., Hua Y., Li X., Zhang C., Chen Y. (2020). Antioxidant and antibacterial activity and in vitro digestion stability of cottonseed protein hydrolysates. LWT.

[9] He Z., Zhang D., Mattison C.P. (2022). Quantitative comparison of the storage protein distribution in glandless and glanded cottonseeds. Agric. Environ. Lett..

[10] He Z., Liu S., Nam S., Klasson K.T., Cheng H.N. (2022). Molecular level characterization of the effect of roasting on the extractable components of glandless cottonseed by Fourier transform ion cyclotron resonance mass spectrometry. Food Chem..

[11] Randel R.D., Chase C.C., Wyse S.J. (1992). Effects of gossypol and cottonseed products on reproduction of mammals. J. Anim. Sci..

[12] Zhang L.M., Zhang Y.Z., Liu Y.Q., Gong Z.H., Zhao Y.M., Li Y.F. (2009). CTN-986, a compound extracted from cottonseeds, increases cell proliferation in hippocampus in vivo and in cultured neural progenitor cells in vitro. Eur. J. Pharmacol..

[13] Cao H., Sethumadhavan K. (2018). Cottonseed extracts and gossypol regulate diacylglycerol acyltransferase gene expression in mouse macrophages. J. Agric. Food Chem..

[14] Kong X., Song W., Hua Y., Li X., Chen Y., Zhang C., Chen Y. (2020). Insights into the antibacterial activity of cottonseed protein-derived peptide against Escherichia coli. Food Funct..

[15] Wang L., Ma M., Yu Z., Du S.K. (2021). Preparation and identification of antioxidant peptides from cottonseed proteins. Food Chem..

[16] Cao H., Qin B., Panickar K.S., Anderson R.A. (2008). Tea and cinnamon polyphenols improve the metabolic syndrome. Agro Food Ind. Hi-Tech.

[17] Hu Q., Liao W., Zhang Z., Shi S., Hou S., Ji N., Zhang X., Zhang Q., Liao Y., Li L. (2022). The hepatoprotective effects of plant-based foods based on the “gut-liver axis”: A prospective review. Crit. Rev. Food Sci. Nutr..

[18] Hazafa A., Rehman K.U., Jahan N., Jabeen Z. (2020). The Role of Polyphenol (Flavonoids) Compounds in the Treatment of Cancer Cells. Nutr. Cancer.

[19] Long J., Guan P., Hu X., Yang L., He L., Lin Q., Luo F., Li J., He X., Du Z. (2021). Natural Polyphenols as Targeted Modulators in Colon Cancer: Molecular Mechanisms and Applications. Front. Immunol..

[20] Mileo A.M., Nistico P., Miccadei S. (2019). Polyphenols: Immunomodulatory and Therapeutic Implication in Colorectal Cancer. Front. Immunol..

[21] Kenar J.A. (2006). Reaction chemistry of gossypol and its derivatives. J. Am. Oil Chem. Soc..

[22] Liu S., Kulp S.K., Sugimoto Y., Jiang J., Chang H.L., Dowd M.K., Wan P., Lin Y.C. (2002). The (-)-enantiomer of gossypol possesses higher anticancer potency than racemic gossypol in human breast cancer. Anticancer Res..

[23] Messeha S.S., Zarmouh N.O., Mendonca P., Alwagdani H., Cotton C., Soliman K.F.A. (2019). Effects of gossypol on apoptosis-related gene expression in racially distinct triple-negative breast cancer cells. Oncol. Rep..

[24] Zhong S., Leong J., Ye W., Xu P., Lin S.H., Liu J.Y., Lin Y.C. (2013). (-)-Gossypol-enriched cottonseed oil inhibits proliferation and adipogenesis of human breast pre-adipocytes. Anticancer Res..

[25] Chien C.C., Ko C.H., Shen S.C., Yang L.Y., Chen Y.C. (2012). The role of COX-2/PGE2 in gossypol-induced apoptosis of colorectal carcinoma cells. J. Cell Physiol..

[26] Cao H., Sethumadhavan K., Cao F., Wang T.T.Y. (2021). Gossypol decreased cell viability and down-regulated the expression of a number of genes in human colon cancer cells. Sci. Rep..

[27] Yuan Y., Tang A.J., Castoreno A.B., Kuo S.Y., Wang Q., Kuballa P., Xavier R., Shamji A.F., Schreiber S.L., Wagner B.K. (2013). Gossypol and an HMT G9a inhibitor act in synergy to induce cell death in pancreatic cancer cells. Cell Death Dis..

[28] Thakur A., Lum L.G., Schalk D., Azmi A., Banerjee S., Sarkar F.H., Mohommad R. (2012). Pan-Bcl-2 inhibitor AT-101 enhances tumor cell killing by EGFR targeted T cells. PLoS ONE.

[29] Pang X., Wu Y., Wu Y., Lu B., Chen J., Wang J., Yi Z., Qu W., Liu M. (2011). (-)-Gossypol suppresses the growth of human prostate cancer xenografts via modulating VEGF signaling-mediated angiogenesis. Mol. Cancer Ther..

[30] Huang Y.W., Wang L.S., Dowd M.K., Wan P.J., Lin Y.C. (2009). (-)-Gossypol reduces invasiveness in metastatic prostate cancer cells. Anticancer Res..

[31] Huo M., Gao R., Jiang L., Cui X., Duan L., Deng X., Guan S., Wei J., Soromou L.W., Feng H. (2013). Suppression of LPS-induced inflammatory responses by gossypol in RAW 264.7 cells and mouse models. Int. Immunopharmacol..

[32] Mellon J.E., Zelaya C.A., Dowd M.K., Beltz S.B., Klich M.A. (2012). Inhibitory effects of gossypol, gossypolone, and apogossypolone on a collection of economically important filamentous fungi. J. Agric. Food Chem..

[33] Prior R.L., Gu L. (2005). Occurrence and biological significance of proanthocyanidins in the American diet. Phytochemistry.

[34] Cao H., Hininger-Favier I., Kelly M.A., Benaraba R., Dawson H.D., Coves S., Roussel A.M., Anderson R.A. (2007). Green tea polyphenol extract regulates the expression of genes involved in glucose uptake and insulin signaling in rats fed a high fructose diet. J. Agric. Food Chem..

[35] Cao H., Kelly M.A., Kari F., Dawson H.D., Urban J.F., Coves S., Roussel A.M., Anderson R.A. (2007). Green tea increases anti-inflammatory tristetraprolin and decreases pro-inflammatory tumor necrosis factor mRNA levels in rats. J. Inflamm..

[36] Cao H., Anderson R.A. (2011). Cinnamon polyphenol extract regulates tristetraprolin and related gene expression in mouse adipocytes. J. Agric. Food Chem..

[37] Cao H., Graves D.J., Anderson R.A. (2010). Cinnamon extract regulates glucose transporter and insulin-signaling gene expression in mouse adipocytes. Phytomedicine.

[38] Cao H., Polansky M.M., Anderson R.A. (2007). Cinnamon extract and polyphenols affect the expression of tristetraprolin, insulin receptor, and glucose transporter 4 in mouse 3T3-L1 adipocytes. Arch. Biochem. Biophys..

[39] Cao H., Urban J.F., Anderson R.A. (2008). Cinnamon polyphenol extract affects immune responses by regulating anti- and proinflammatory and glucose transporter gene expression in mouse macrophages. J. Nutr..

[40] Cao H., Sethumadhavan K., Li K., Boue S.M., Anderson R.A. (2019). Cinnamon polyphenol extract and insulin regulate diacylglycerol acyltransferase gene expression in mouse adipocytes and macrophages. Plant Foods Hum. Nutr..

[41] Cao H., Sethumadhavan K. (2019). Gossypol but not cottonseed extracts or lipopolysaccharides stimulates HuR gene expression in mouse cells. J. Funct. Foods.

[42] Cao H., Sethumadhavan K. (2020). Regulation of cell viability and anti-inflammatory tristetraprolin family gene expression in mouse macrophages by cottonseed extracts. Sci. Rep..

[43] Rietschel E.T., Kirikae T., Schade F.U., Mamat U., Schmidt G., Loppnow H., Ulmer A.J., Zähringer U., Seydel U., Di Padova F. (1994). Bacterial endotoxin: Molecular relationships of structure to activity and function. FASEB J..

[44] Bustin S.A. (2002). Quantification of mRNA using real-time reverse transcription PCR (RT-PCR): Trends and problems. J. Mol. Endocrinol..

[45] Cao H., Cao F., Klasson K.T. (2013). Characterization of reference gene expression in tung tree (*Vernicia fordii*). Ind. Crops Prod..

[46] Bustin S.A., Benes V., Garson J.A., Hellemans J., Huggett J., Kubista M., Mueller R., Nolan T., Pfaffl M.W., Shipley G.L. (2009). The MIQE guidelines: Minimum information for publication of quantitative real-time PCR experiments. Clin. Chem..

[47] Bustin S.A., Benes V., Nolan T., Pfaffl M.W. (2005). Quantitative real-time RT-PCR—A perspective. J. Mol. Endocrinol..

[48] Livak K.J., Schmittgen T.D. (2001). Analysis of relative gene expression data using real-time quantitative PCR and the 2^−∆∆C^_T_ Method. Methods.

[49] Pfaffl M.W. (2001). A new mathematical model for relative quantification in real-time RT-PCR. Nucleic Acids Res..

[50] Udvardi M.K., Czechowski T., Scheible W.R. (2008). Eleven golden rules of quantitative RT-PCR. Plant Cell.

[51] Lejeune P., Reisser D., Onier N., Lagadec P., Lindley I., Jeannin J.F. (1994). Interleukin-8 has antitumor effects in the rat which are not associated with polymorphonuclear leukocyte cytotoxicity. Cancer Immunol. Immunother..

[52] Yoshioka T., Morimoto Y., Iwagaki H., Itoh H., Saito S., Kobayashi N., Yagi T., Tanaka N. (2001). Bacterial lipopolysaccharide induces transforming growth factor beta and hepatocyte growth factor through toll-like receptor 2 in cultured human colon cancer cells. J. Int. Med. Res..

[53] Ikebe M., Kitaura Y., Nakamura M., Tanaka H., Yamasaki A., Nagai S., Wada J., Yanai K., Koga K., Sato N. (2009). Lipopolysaccharide (LPS) increases the invasive ability of pancreatic cancer cells through the TLR4/MyD88 signaling pathway. J. Surg.Oncol..

[54] Liu W.T., Jing Y.Y., Yan F., Han Z.P., Lai F.B., Zeng J.X., Yu G.F., Fan Q.M., Li R., Zhao Q.D. (2017). LPS-induced CXCR4-dependent migratory properties and a mesenchymal-like phenotype of colorectal cancer cells. Cell Adhes. Migr..

[55] Lee H.H., Kim W.T., Kim D.H., Park J.W., Kang T.H., Chung J.W., Leem S.H. (2013). Tristetraprolin suppresses AHRR expression through mRNA destabilization. FEBS Lett..

[56] Kitada S., Kress C.L., Krajewska M., Jia L., Pellecchia M., Reed J.C. (2008). Bcl-2 antagonist apogossypol (NSC736630) displays single-agent activity in Bcl-2-transgenic mice and has superior efficacy with less toxicity compared with gossypol (NSC19048). Blood.

[57] Frevel M.A., Bakheet T., Silva A.M., Hissong J.G., Khabar K.S., Williams B.R. (2003). p38 Mitogen-activated protein kinase-dependent and -independent signaling of mRNA stability of AU-rich element-containing transcripts. Mol. Cell. Biol..

[58] Xu L., Ning H., Gu L., Wang Q., Lu W., Peng H., Cui W., Ying B., Ross C.R., Wilson G.M. (2015). Tristetraprolin induces cell cycle arrest in breast tumor cells through targeting AP-1/c-Jun and NF-kappaB pathway. Oncotarget.

[59] Sharma A., Bhat A.A., Krishnan M., Singh A.B., Dhawan P. (2013). Trichostatin-A modulates claudin-1 mRNA stability through the modulation of Hu antigen R and tristetraprolin in colon cancer cells. Carcinogenesis.

[60] Warzych E., Wolc A., Cieslak A., Lechniak-Cieslak D. (2012). 217 transcript abundance of cathepsin genes in cumulus cells as a marker of cattle oocyte quality. Reprod. Fertil. Dev..

[61] Sawaoka H., Dixon D.A., Oates J.A., Boutaud O. (2003). Tristetraprolin binds to the 3’-untranslated region of cyclooxygenase-2 mRNA. A polyadenylation variant in a cancer cell line lacks the binding site. J. Biol. Chem..

[62] Lee W.H., Lee H.H., Vo M.T., Kim H.J., Ko M.S., Im Y.C., Min Y.J., Lee B.J., Cho W.J., Park J.W. (2011). Casein kinase 2 regulates the mRNA-destabilizing activity of tristetraprolin. J. Biol. Chem..

[63] Fuhrmann D.C., Tausendschon M., Wittig I., Steger M., Ding M.G., Schmid T., Dehne N., Brune B. (2015). Inactivation of tristetraprolin in chronic hypoxia provokes the expression of cathepsin B. Mol. Cell Biol..

[64] Datta S., Biswas R., Novotny M., Pavicic P.G., Herjan T., Mandal P., Hamilton T.A. (2008). Tristetraprolin regulates CXCL1 (KC) mRNA stability. J. Immunol..

[65] Ligueros M., Jeoung D., Tang B., Hochhauser D., Reidenberg M.M., Sonenberg M. (1997). Gossypol inhibition of mitosis, cyclin D1 and Rb protein in human mammary cancer cells and cyclin-D1 transfected human fibrosarcoma cells. Br. J. Cancer.

[66] Dong Y., Mao B., Li L., Guan H., Su Y., Li X., Lian Q., Huang P., Ge R.S. (2015). Gossypol enantiomers potently inhibit human placental 3beta-hydroxysteroid dehydrogenase 1 and aromatase activities. Fitoterapia.

[67] Ludwig E.H., Mahley R.W., Palaoglu E., Ozbayrakci S., Balestra M.E., Borecki I.B., Innerarity T.L., Farese R.V. (2002). DGAT1 promoter polymorphism associated with alterations in body mass index, high density lipoprotein levels and blood pressure in Turkish women. Clin. Genet..

[68] Dey P., Chakraborty M., Kamdar M.R., Maiti M.K. (2014). Functional characterization of two structurally novel diacylglycerol acyltransferase2 isozymes responsible for the enhanced production of stearate-rich storage lipid in Candida tropicalis SY005. PLoS ONE.

[69] Lee H.H., Lee S.R., Leem S.H. (2014). Tristetraprolin regulates prostate cancer cell growth through suppression of E2F1. J. Microbiol. Biotechnol..

[70] Florkowska M., Tymoszuk P., Balwierz A., Skucha A., Kochan J., Wawro M., Stalinska K., Kasza A. (2012). EGF activates TTP expression by activation of ELK-1 and EGR-1 transcription factors. BMC Mol. Biol..

[71] Chang J.S., Hsu Y.L., Kuo P.L., Chiang L.C., Lin C.C. (2004). Upregulation of Fas/Fas ligand-mediated apoptosis by gossypol in an immortalized human alveolar lung cancer cell line. Clin. Exp. Pharmacol. Physiol..

[72] Chen D., Pan X., Xiao P., Farwell M.A., Zhang B. (2011). Evaluation and identification of reliable reference genes for pharmacogenomics, toxicogenomics, and small RNA expression analysis. J. Cell Physiol..

[73] Fahling M., Persson A.B., Klinger B., Benko E., Steege A., Kasim M., Patzak A., Persson P.B., Wolf G., Bluthgen N. (2012). Multilevel regulation of HIF-1 signaling by TTP. Mol. Biol. Cell.

[74] Kagami S., Kanari H., Suto A., Fujiwara M., Ikeda K., Hirose K., Watanabe N., Iwamoto I., Nakajima H. (2008). HMG-CoA reductase inhibitor simvastatin inhibits proinflammatory cytokine production from murine mast cells. Int. Arch. Allergy Immunol..

[75] Jamal U.M., Joe Y., Zheng M., Blackshear P.J., Ryter S.W., Park J.W., Chung H.T. (2013). A functional link between heme oxygenase-1 and tristetraprolin in the anti-inflammatory effects of nicotine. Free Radic. Biol. Med..

[76] Shi J.X., Li J.S., Hu R., Shi Y., Su X., Li Q., Zhang F. (2014). CNOT7/hCAF1 is involved in ICAM-1 and IL-8 regulation by tristetraprolin. Cell Signal..

[77] Su N.Y., Tsai P.S., Huang C.J. (2008). Clonidine-Induced Enhancement of iNOS Expression Involves NF-kappaB. J. Surg. Res..

[78] Ogilvie R.L., Abelson M., Hau H.H., Vlasova I., Blackshear P.J., Bohjanen P.R. (2005). Tristetraprolin down-regulates IL-2 gene expression through AU-rich element-mediated mRNA decay. J. Immunol..

[79] Hochdorfer T., Tiedje C., Stumpo D.J., Blackshear P.J., Gaestel M., Huber M. (2013). LPS-induced production of TNF-alpha and IL-6 in mast cells is dependent on p38 but independent of TTP. Cell Signal..

[80] Balakathiresan N.S., Bhattacharyya S., Gutti U., Long R.P., Jozwik C., Huang W., Srivastava M., Pollard H.B., Biswas R. (2009). Tristetraprolin regulates IL-8 mRNA stability in cystic fibrosis lung epithelial cells. Am. J. Physiol.-Lung Cell. Mol. Physiol..

[81] Gaba A., Grivennikov S.I., Do M.V., Stumpo D.J., Blackshear P.J., Karin M. (2012). Cutting edge: IL-10-mediated tristetraprolin induction is part of a feedback loop that controls macrophage STAT3 activation and cytokine production. J. Immunol..

[82] Gu L., Ning H., Qian X., Huang Q., Hou R., Almourani R., Fu M., Blackshear P.J., Liu J. (2013). Suppression of IL-12 production by tristetraprolin through blocking NF-kcyB nuclear translocation. J. Immunol..

[83] Milke L., Schulz K., Weigert A., Sha W., Schmid T., Brune B. (2013). Depletion of tristetraprolin in breast cancer cells increases interleukin-16 expression and promotes tumor infiltration with monocytes/macrophages. Carcinogenesis.

[84] Datta S., Novotny M., Pavicic P.G., Zhao C., Herjan T., Hartupee J., Hamilton T. (2010). IL-17 regulates CXCL1 mRNA stability via an AUUUA/tristetraprolin-independent sequence. J. Immunol..

[85] Xu P., Ye W., Zhong S., Li H., Feng E., Lin S.H., Kuo C.T., Liu J.Y., Lin Y.C. (2010). Leptin and zeranol up-regulate cyclin D1 expression in primary cultured normal human breast pre-adipocytes. Mol. Med. Rep..

[86] Voss A.K., Thomas T., Gruss P. (1998). Compensation for a gene trap mutation in the murine microtubule-associated protein 4 locus by alternative polyadenylation and alternative splicing. Dev. Dyn..

[87] Jiang J., Slivova V., Jedinak A., Sliva D. (2012). Gossypol inhibits growth, invasiveness, and angiogenesis in human prostate cancer cells by modulating NF-kappaB/AP-1 dependent- and independent-signaling. Clin. Exp. Metastasis.

[88] Barba-Barajas M., Hernandez-Flores G., Lerma-Diaz J.M., Ortiz-Lazareno P.C., Dominguez-Rodriguez J.R., Barba-Barajas L., de Celis R., Jave-Suarez L.F., Aguilar-Lemarroy A.C., Guevara-Barraza M.G. (2009). Gossypol induced apoptosis of polymorphonuclear leukocytes and monocytes: Involvement of mitochondrial pathway and reactive oxygen species. Immunopharmacol. Immunotoxicol..

[89] Kim H.K., Kim C.W., Vo M.T., Lee H.H., Lee J.Y., Yoon N.A., Lee C.Y., Moon C.H., Min Y.J., Park J.W. (2012). Expression of proviral integration site for Moloney murine leukemia virus 1 (Pim-1) is post-transcriptionally regulated by tristetraprolin in cancer cells. J. Biol. Chem..

[90] Huang Y.W., Wang L.S., Chang H.L., Ye W., Dowd M.K., Wan P.J., Lin Y.C. (2006). Molecular mechanisms of (-)-gossypol-induced apoptosis in human prostate cancer cells. Anticancer Res..

[91] Militello R.D., Munafo D.B., Beron W., Lopez L.A., Onier S., Oud B., Olombo M.I., Oloris S.C. (2013). Rab24 is Required for Normal Cell Division. Traffic.

[92] Brattelid T., Winer L.H., Levy F.O., Liestol K., Sejersted O.M., Andersson K.B. (2010). Reference gene alternatives to Gapdh in rodent and human heart failure gene expression studies. BMC Mol. Biol..

[93] Yeow W.S., Baras A., Chua A., Nguyen D.M., Sehgal S.S., Schrump D.S., Nguyen D.M. (2006). Gossypol, a phytochemical with BH3-mimetic property, sensitizes cultured thoracic cancer cells to Apo2 ligand/tumor necrosis factor-related apoptosis-inducing ligand. J. Thorac. Cardiovasc. Surg..

[94] Gao W., Shen Z., Shang L., Wang X. (2011). Upregulation of human autophagy-initiation kinase ULK1 by tumor suppressor p53 contributes to DNA-damage-induced cell death. Cell Death. Differ..

[95] Essafi-Benkhadir K., Onesto C., Stebe E., Moroni C., Pages G. (2007). Tristetraprolin inhibits ras-dependent tumor vascularization by inducing VEGF mRNA degradation. Mol. Biol. Cell.

[96] He G., Sun D., Ou Z., Ding A. (2012). The protein Zfand5 binds and stabilizes mRNAs with AU-rich elements in their 3’-untranslated regions. J. Biol. Chem..

[97] Cao H., Shockey J.M. (2012). Comparison of TaqMan and SYBR Green qPCR methods for quantitative gene expression in tung tree tissues. J. Agric. Food Chem..

[98] Yun E.J., Yu S., Kim Y.A., Liu J.J., Kang N.J., Jin Y.S., Kim K.H. (2021). In Vitro Prebiotic and Anti-Colon Cancer Activities of Agar-Derived Sugars from Red Seaweeds. Mar. Drugs.

[99] Zhao X., Song J.L., Kim J.D., Lee J.S., Park K.Y. (2013). Fermented Pu-erh tea increases in vitro anticancer activities in HT-29 cells and has antiangiogenetic effects on HUVECs. J. Environ. Pathol. Toxicol. Oncol..

[100] Gu Q., Hu C., Chen Q., Xia Y. (2013). Tea polyphenols prevent lung from preneoplastic lesions and effect p53 and bcl-2 gene expression in rat lung tissues. Int. J. Clin. Exp. Pathol..

[101] Sandur S.K., Deorukhkar A., Pandey M.K., Pabon A.M., Shentu S., Guha S., Aggarwal B.B., Krishnan S. (2009). Curcumin modulates the radiosensitivity of colorectal cancer cells by suppressing constitutive and inducible NF-kappaB activity. Int. J. Radiat. Oncol. Biol. Phys..

[102] Su C.C., Lin J.G., Li T.M., Chung J.G., Yang J.S., Ip S.W., Lin W.C., Chen G.W. (2006). Curcumin-induced apoptosis of human colon cancer colo 205 cells through the production of ROS, Ca2+ and the activation of caspase-3. Anticancer Res..

[103] Laka K., Mapheto K.B.F., Mbita Z. (2021). Selective in vitro cytotoxicity effect of Drimia calcarata bulb extracts against p53 mutant HT-29 and p53 wild-type Caco-2 colorectal cancer cells through STAT5B regulation. Toxicol. Rep..

[104] Dai W., Mu L., Cui Y., Li Y., Chen P., Xie H., Wang X. (2019). Berberine Promotes Apoptosis of Colorectal Cancer via Regulation of the Long Non-Coding RNA (lncRNA) Cancer Susceptibility Candidate 2 (CASC2)/AU-Binding Factor 1 (AUF1)/B-Cell CLL/Lymphoma 2 (Bcl-2) Axis. Med. Sci. Monit..

[105] Ko C.H., Shen S.C., Yang L.Y., Lin C.W., Chen Y.C. (2007). Gossypol reduction of tumor growth through ROS-dependent mitochondria pathway in human colorectal carcinoma cells. Int. J. Cancer.

[106] Lan L., Appelman C., Smith A.R., Yu J., Larsen S., Marquez R.T., Liu H., Wu X., Gao P., Roy A. (2015). Natural product (-)-gossypol inhibits colon cancer cell growth by targeting RNA-binding protein Musashi-1. Mol.Oncol..

[107] Hu Z.Y., Wang J., Cheng G., Zhu X.F., Huang P., Yang D., Zeng Y.X. (2011). Apogossypolone targets mitochondria and light enhances its anticancer activity by stimulating generation of singlet oxygen and reactive oxygen species. Chin. J. Cancer.

[108] Yan F., Cao X.X., Jiang H.X., Zhao X.L., Wang J.Y., Lin Y.H., Liu Q.L., Zhang C., Jiang B., Guo F. (2010). A novel water-soluble gossypol derivative increases chemotherapeutic sensitivity and promotes growth inhibition in colon cancer. J. Med. Chem..

[109] Cao H., Cao F., Roussel A.M., Anderson R.A. (2013). Quantitative PCR for glucose transporter and tristetraprolin family gene expression in cultured mouse adipocytes and macrophages. In Vitro Cell Dev. Biol. Anim..

